# Egg quality and laying performance of Julia laying hens fed with black soldier fly (*Hermetia illucens*) larvae meal as a long-term substitute for fish meal

**DOI:** 10.1016/j.psj.2022.101986

**Published:** 2022-06-02

**Authors:** Junliang Zhao, Kiyonori Kawasaki, Hironori Miyawaki, Hirofumi Hirayasu, Akihisa Izumo, Shun-ichiro Iwase, Koji Kasai

**Affiliations:** ⁎ Faculty of Agriculture, Kagawa University, Kagawa, 761-0795, Japan; † Research Institute of Environment, Agriculture and Fisheries, Osaka Prefecture, Osaka, 583-0862, Japan

**Keywords:** black soldier fly larvae meal, egg quality, fish meal, laying hens’ performance, leftover

## Abstract

The use of insects in animal feed appears to be an efficient approach that contributes to solving the environmental issues related to leftover disposal; however, it has not been approved in some countries due to concerns about pathogenic infections. This study aimed to evaluate the feasibility of long-term substitution of fish meal in poultry feed with organic defatted black soldier fly larvae (**BSFL**) meal prepared from BSFL raised on leftovers. The 87 Julia laying hens (178-day-old) were allotted in a completely randomized design with three treatments (29 layers in each treatment). The laying hens were fed maize grain and soybean meal-based diet containing either 3% fish meal, 1.5% fish meal and 1.5% BSFL meal, or 3% BSFL meal supplements for 52 wk (541-day-old). Results showed that substituting fish meal with BSFL meal had no effect on the laying rate, feed intake, and feed conversion ratio of laying hens, and only the complete replacement (3% BSFL meal) significantly increased the body weight of laying hens. In terms of egg quality, there was no significant effect on eggshell parameters (weight, thickness, and strength), albumen weight, yolk height, yolk color, and Haugh unit. However, both half (1.5% fish meal and 1.5% BSFL meal) and complete substitution of fish meal increased yolk weight (*P* < 0.01) and egg weight (*P* < 0.05). In conclusion, even if BSFL were fed leftovers and the meal was defatted with organic solvents, it can be used as a poultry feed ingredient without any adverse effect. Moreover, the complete substitution of fish meal with BSFL meal may be a feasible way to effectively contribute to the laying hens’ performances and poultry farming costs. In addition to fish meal, the replacement of soybean meal with BSFL meal may also needs to be further studied for the extensive BSFL meal application in poultry feed.

## INTRODUCTION

The global human population increment seems to challenge the future food supply chain. Simultaneously, overpopulation may also put extraordinary pressure on our ecosystem (e.g., land, water, and air) (Garg, 2017). The Food and Agricultural Organization of the United Nations has predicted that global food production may need to increase by 50% if the population is over 9 billion by 2050 (FAO, 2017). The utilization of insects in animal feed is widely considered to contribute to sustainable development not only by meeting the growing demand of animal feed and animal products’ consumption, but also the land-sparing that is proposed to increase the yield with limited farmland. The European Union had recently approved insect-processed animal protein usage in poultry and pig feed to advance the industrialization of insect meal (Commission Regulation (EU), 2021).

The black soldier fly (*Hermetia illucens*) is a species that has been commercially exploited by several companies as it can be easily reared on most organic wastes, such as livestock wastes, food manufacture residues, and household wastes in a cost-effective and ecofriendly manner (Hopkins et al., 2021; Kawasaki et al., 2021; Siddiqui et al., 2022). In addition, black soldier fly larvae (**BSFL**) can decompose and convert organic wastes into nutritious feed ingredients that are rich in protein, some essential amino acids, fatty acids, and micronutrients (Wang and Shelomi, 2017; Cullere et al., 2018; Shumo et al*.*, 2019). The substrates used for culturing BSFL influence the nutritional content of the BSFL meal. For instance, using brewery by-products as a substrate contributes to BSFL meal with a high proportion of protein (Liu et al., 2018), while using fruits and vegetables as substrates results in a low protein proportion (Barbi et al., 2020). The crude protein content of BSFL meal varies broadly from 12.9 to 78.8% on a dry matter basis, as does the fat content, which ranges from 2.2 to 57.8% (Hopkins et al., 2021). Nevertheless, all values reinforce the fact that BSFL meal can be a low-energy, intensive protein and fat source in animal feed. Thus, including BSFL meals with such different nutritional compositions in animal feed may positively influence animals’ performance.

A few studies have analyzed the effect of using BSFL meal as a protein replacement in poultry feed. For instance, the proportional BSFL meal substitution for soybean meal in daily rations of broiler quails showed a comparable effect with the control on overall apparent nutrient digestibility and productive efficiency (Cullere et al., 2016; Mat et al., 2021). Similarly, no adverse effect was observed when soybean meal in layer diets was partially and completely replaced with BSFL meal (Park et al., 2017; Heuel et al., 2021a). Therefore, BSFL meal is considered a feasible alternative to soybean meal in poultry feed. Unlike ruminant livestock, poultry lacks the complex protein synthesis metabolism; therefore, incorporating fish meal in addition to soybean meal in poultry feed helps to meet the requirements of certain minerals (calcium and phosphorus) and essential amino acids (methionine and lysine; Asghedom et al., 2006). Unfortunately, fish meal sources are unsustainable, costly, and at risk of the presence of high-level histamine and foodborne pathogens (Shepherd and Jackson, 2013). Such issues make insects, like BSFL, alternative sources for total or partial replacement of fish meal. Furthermore, a study on the substitution of fish meal with BSFL meal in aquaculture feed showed that BSFL meal does not adversely affect feed efficiency, meat sensory properties, and mucosal immune response (Tippayadara et al., 2021). However, data on the effect of fish meal replacement with BSFL in laying hen feed is limited and inconclusive. In addition, although the use of BSFL meal in animal feed has been authorized (Commission Regulation (EU), 2021), some larval rearing substrates, such as household waste and leftovers, are still unapproved in some countries. Thus, larger-scale trials with extensive feeding strategies are needed. In a previous study, we had indicated that household organic wastes could be used as a substrate for feeding BSFL, and the obtained BSFL and pre-pupae were a potential alternate for soybean meal and oil in poultry feed without any adverse effect (Kawasaki et al*.*, 2019). In the present study, we aimed to evaluate if BSFL reared on leftovers and defatted with organic solvents could be an alternative to fish meal in poultry feed by assaying the layer performances and egg qualities, so as to extend BSFL meal utilization and reduce the cost of poultry feed.

## MATERIALS AND METHODS

### Black Soldier Fly Larvae Meal Preparation

The BSFL meal was prepared according to the method described previously (Kawasaki et al*.*, 2019), with a minor modification. Briefly, the collected black soldier fly eggs were raised with commercial poultry feed (Nichiwa Sangyo Co., Ltd., Hyogo, Japan) for a week, then the hatched larvae were transferred to a rearing substrate consisting of cabbage, rice, and chicken in 65:20:15 ratio, and bred for 8 d at 25 to 30°C. After that, the obtained BSFL were heat-killed at 90°C for 30 s and dried at 45°C for 48 h. Subsequently, the dried BSFL were pulverized and defatted (solvent: 90% Hexane and 10% ethanol) before adding to laying hens’ diets.

### Animal Experiment Design and Diets

The animal experiment was conducted under the approval of the Kagawa University Animal Experiment Committee (Permission number: 2019-19666) and the laws of the Japanese Association of Laboratory Animal Facilities of National University Corporation. The 87 Julia layers at 126 days old were obtained from a commercial rearing farm (Niinobe, Kagawa, Japan) with the same husbandry management (free access to feed and water, lights on for 11 h) and subjected to adaptation for 45 d until getting 80% of laying rate. Thereafter, the 87 laying hens were assigned into 3 treatments with similar average body weight and continued adaptation for 1 wk. The laying hens were housed in an evaporation room with individual cages (45 cm length × 40 cm height × 22 cm width) under uncontrolled ambient temperature (about 13°C to 32°C) and humidity (about 55 to 85%), and 12 h light/dark cycle. Each cage was equipped with a feed container and a nipple drinker. Each treatment contains three replicates (9, 9, and 11 laying hens). All layers had ad libitum access to the diets and water, following the daily nutrient requirements based on the recommendations of the National Agriculture and Food Research Organization (JLIA, 2011). The feeding trial was started using 178-days-age layers and was performed in November 2019 and for 52 wk.

Maize grain and soybean meal-based diet supplemented with fish meal, BSFL meal, vitamins, minerals, and amino acids were formulated to evaluate the effect of substituting fish meal with BSFL meal on layers. Diet free of BSFL meal was served as the control (3% fish meal), while diets with half or complete substitution of fish meal with BSFL meal, which accounted for 1.5% and 3.0% of dry basis, respectively, were treated as the experimental groups. Proximate nutrients of fish meal, BSFL meal, and diets were analyzed according to the methods of the Association of Official Analytical Chemists (AOAC, 1990). The proximate nutrients and energy content of diets are described in Table 1.

**Table 1:** Ingredients and proximate nutrients of the experimental diets.

Ingredients (% in fresh matter)	Control diet	1.5% BSFL meal diet	3% BSFL meal diet
Maize grain	59.0	59.0	59.0
Soybean meal	21.0	21.0	21.0
Calcium carbonate	8.0	8.0	8.0
Rice bran	4.0	4.0	4.0
Fish meal	3.0	1.5	0.0
BSFL meal	0.0	1.5	3.0
Corn oil	2.0	2.0	2.0
Salt	1.0	1.0	1.0
Tricalcium phosphate	1.0	1.0	1.0
Vitamin and mineral mix^1^	1.0	1.0	1.0
Proximate nutrients (% in dry matter), minerals and energy content^2^			
Water content (% in fresh matter)	12.0	12.0	12.0
Ca^3^ (g/kg)	3.7	3.6	3.5
P^3^ (g/kg)	0.4	0.4	0.4
Ca/P^3^	9.3	9.0	8.8
Crude protein^3^	16.4	16.1	15.8
Crude protein^4^	19.1	19.1	19.8
Crude fat^4^	6.2	6.2	6.5
Crude fiber^4^	3.7	3.4	3.4
Crude ash^4^	12.4	12.2	12.6
NFE^3^	46.6	47.0	45.7
ME (Mcal/kg)^3^^,^^5^	3.27	3.29	3.29

Abbreviations: BSFL, Black Soldier Fly Larvae; NFE, nitrogen free extract.

1 Vitamin and mineral mix: water (3.4%); Na (300.00 g/kg); Fe (8.20 g/kg); Zn (2.20 g/kg); Mn (10.00 g/kg); Cu (0.62 g/kg); choline (30.00 g/kg); vitamin A (3,000,000 IU/kg); vitamin B_1_ (0.14 g/kg); vitamin B_2_ (0.35 g/kg); vitamin B_3_ (1.40 g/kg); vitamin B_5_ (0.50 g/kg); vitamin B_6_ (0.25 g/kg); vitamin B_7_ (0.50 g/kg); vitamin B_9_ (0.025 g/kg); vitamin B_12_ (0.0005 g/kg); vitamin D (600,000 IU/kg); vitamin E (596 IU/kg).

2 Water content, Ca, P, and ME were presented in fresh matter, while the other proximate nutrients were analyzed in dry matter.

3 Calculated data.

4 Analyzed data.

5 ME = (Crude protein × 3.84 + Crude fat × 9.33 + NFE × 4.2) / 100.

### Total Amino Acid Composition Analysis of Fish Meal and BSFL Meal

Total amino acid composition analysis of fish and BSFL meals was performed using liquid chromatography-mass spectrometry. A 5 mg sample was hydrolyzed according to the description of the Analytical Manual for the Standard Tables of Food Composition in Japan (MEXT, 2015). Then, after centrifugation, the obtained supernatant was filtered with a membrane filter (0.45 μm, RephiLe Bioscience, Japan) and applied into LCMS-2020 ultra-fast liquid chromatography system (Shimadzu, Kyoto, Japan) with an electrospray ionization interface. The column used for analysis was the Intrada Amino Acid column (100 × 3 mm, 3-μm particle size, Imatakt, Japan), and the mobile phase was composed of solvent A (20% acetonitrile in 100 mM ammonium formate solution) and solvent B (acetonitrile/tetrahydrofuran/25 mM ammonium formate/formic acid  =  9/75/16/0.3). Elution was carried out using a gradient mobile buffer (0-17-100% of mobile phase A) that increased from 3-6.5-10 min at 40°C with a flow rate of 0.6 mL/min. Data collection and analyses were performed using LabSolutions LCMS software Ver.5.6 (Shimadzu, Kyoto, Japan).

### Laying Performance Determination

Eggs were collected daily, and the number of eggs and total egg weight were recorded. The laying rate of each group was calculated each week as the total number of eggs divided by days. The feed intake and feed conversion ratio (**FCR**) were also accounted for every week, while individual body weight on every 4-wk basis. The FCR was reported as feed consumption (g) divided by egg weight (g). Data including laying rate, feed intake, and FCR were reported as the average value of 4 wk, and the data collected from the layers that died during the experiment were excluded.

### Egg Quality Analysis

During the 52-wk feeding trial, the egg weight was reported as the average weight value of all eggs in each group. Twelve replicate eggs that were close to the average egg weight of each group were collected for quality analysis. The eggshell parameters (weight, thickness, and strength), albumen weight, Haugh unit, and yolk parameters (weight, height, and color) were evaluated. Eggshell thickness was assessed using an eggshell thickness gauge (Eggshell thickness gauge, Fujihira Industry Co., Ltd., Tokyo, Japan). Eggshell strength was measured with an automatic egg strength meter (Egg Shell Strength Meter, Fujihira Industry Co., Ltd.). The eggshell weight, albumen weight, Haugh unit, and yolk parameters were evaluated using an automatic egg quality multitester (EMT-5000, JA ZEN-NOH EGG Co., Ltd., Tokyo, Japan).

### Statistical Analysis

All data analyses were conducted using the IBM SPSS statistical package (Version: SPSS 26.0, IBM Corp, NY). Shapiro-wilk test was applied for normal distribution check. Levene's test was used for homogeneity of variance test. Repeated measures ANOVA was used for significance analysis of layers’ performance and egg quality among different groups and weeks. The group was set as a between subject factor, while week was set as a within subject factor. Kruskal-Wallis H test was used for motality analysis. Games-Howell Post-Hoc test was used when homogeneity of variance test was violated (body weight and egg weight analysis). Tukey's honest significant difference Post-Hoc test was used for equal sample sizes (egg quality analysis), while Tukey-Krammer Post-Hoc test was applied for unequal sample sizes (laying rate, feed intake, and feed conversion ratio analysis). The statistical significance was considered at a *P-*value less than 0.05.

## RESULTS

### Proximate Nutrients and Amino Acids of Fish Meal and BSFL Meal

The proximate nutrients of fish meal and BSFL meal, including crude protein, crude fat, crude ash, crude fiber, and amino acid composition, are shown in Table 2. The crude protein and crude ash in BSFL meal were lower than fish meal, while crude fat and crude fiber were higher than fish meal. In addition, the amino acid proportion in BSFL was higher in tyrosine, proline, serine and alanine, lower in lysine, glycine, arginine, methionine, and isoleucine, and comparable in aspartic acid, cysteine, glutamic acid, leucine, phenylalanine, threonine tryptophan, and valine, compared to the fish meal.

**Table 2:** Proximate nutrients and amino acid contents of fish meal and BSFL meal.

Proximate nutrients (% in dry matter)^1^	Fish meal	BSFL meal
Crude protein	62.7	52.6
Crude fat	9.0	15.0
Crude ash	19.1	6.5
Crude fiber	0.4	6.4
Amino acid composition (w/w% in total amino acid)^1^		
Arginine	6.5	5.3
Alanine	7	8.0
Aspartic acid	9.6	9.8
Cysteine	1	1.1
Glutamic acid	13.1	13.1
Glycine	7.8	6.0
Histidine	3.3	3.0
Isoleucine	4.3	3.2
Leucine	8.1	7.5
Lysine	8.3	6.1
Methionine	3.1	1.9
Phenylalanine	4.3	4.0
Proline	5	6.7
Serine	4.3	5.5
Threonine	4.6	4.2
Tryptophan	1.3	1.7
Tyrosine	3.3	7.1
Valine	5.1	5.7

Abbreviation: BSFL, Black Soldier Fly Larvae.

1 Analyzed data.

### Laying Hen Performance

**Figure. 1:**
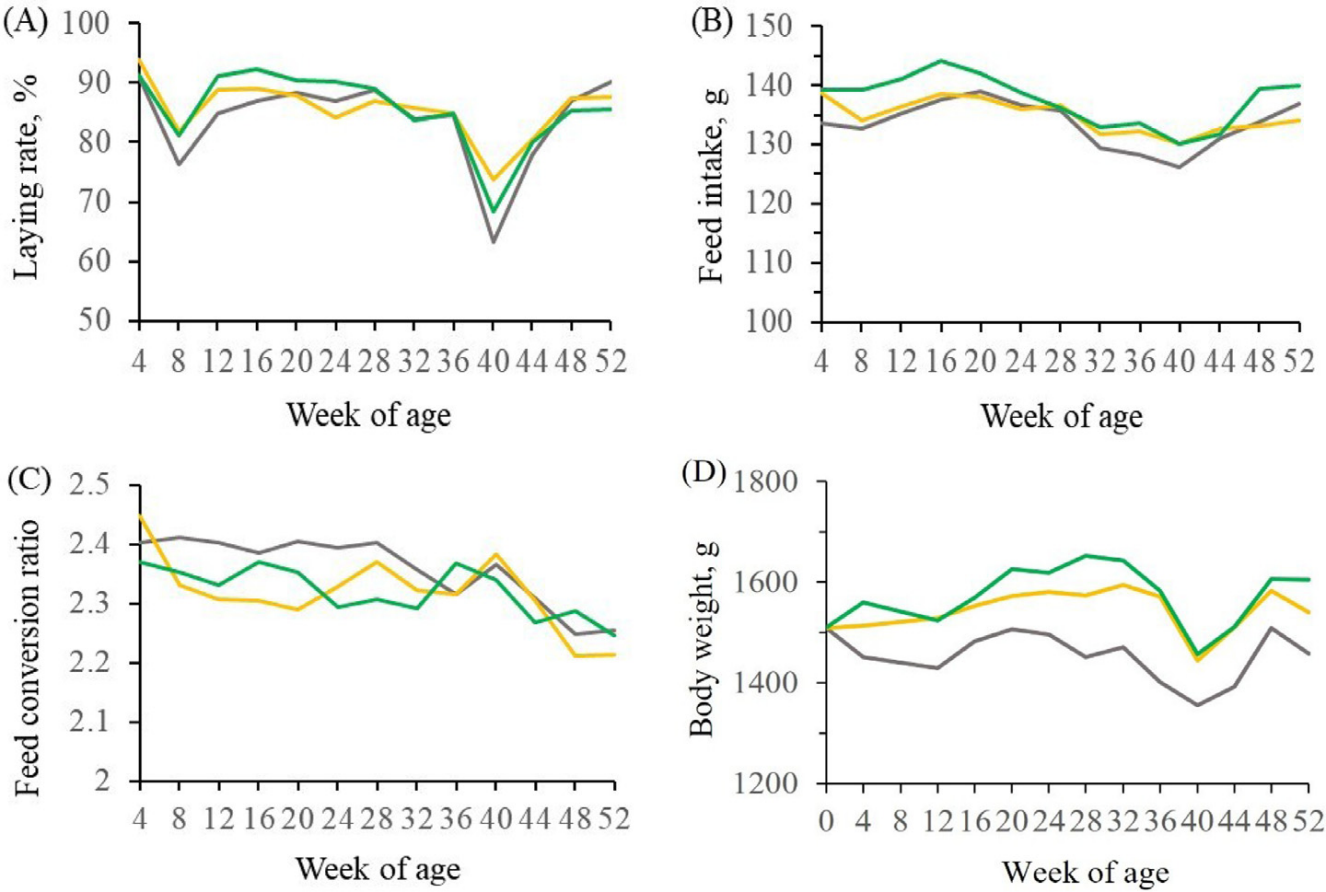
The transition of laying rate, feed intake, feed conversion ratio, and body weight of the laying hens with different proportions of BSFL meal substitution throughout the experiment. (A) Laying rate; (B) feed intake; (C) feed conversion ratio; (D) body weight. The control diet (); 1.5% BSFL meal diet (); 3.0% BSFL meal diet (). Data are reported as the mean of the performance of all individuals in each group over 4 wk. Abbreviation: BSFL, Black Soldier Fly Larvae.

The laying rate, feed intake, body weight, FCR, and mortality of the layers were assayed during the 52-wk feeding trial (Figure 1 and Table 3). The laying rate fluctuation of the BSFL substitutional groups was similar to that of the control. The average laying rate of the BSFL substitutional groups was slightly higher, but the difference was not significant. The weekly transition lines of feed intake and FCR of BSFL substitutional groups were close to that of the control, and there was no significant difference among the groups. In addition, the layers in the BSFL substitutional groups showed a higher weight in the feeding trial. Compared to the control group, there was an increase (*P* < 0.01) in weight in the 3.0% BSFL meal substitutional group, but not in the 1.5% BSFL meal substitutional group. The performances of laying hens significantly differed at 4-wk intervals, but not with the interaction of weeks and groups. The mortality of laying hens in all groups did not show statistical significance.

**Table 3:** Effect of dietary treatments with different proportion of BSFL meal substitution on performance of laying hens^1^.

Item	Control diet	1.5% BSFL meal diet	3.0% BSFL meal diet	*P*-values of effects		
				Group	Period (4 wk)	Groups × Period
Laying rate (%)^2^	83.84 ± 5.547	85.61 ± 11.261	83.84 ± 5.647	0.412	< 0.001	0.654
Feed intake (g)^2^	133.48 ± 4.955	134.59 ± 3.969	137.28 ± 1.661	0.495	< 0.001	0.456
Feed conversation ratio^2^	2.36 ± 0.097	2.31 ± 0.079	2.32 ± 0.020	0.133	< 0.001	0.756
Body weight (g)^2^	133.48 ± 4.955^a^	134.59 ± 3.969^ab^	137.28 ± 1.661^b^	0.012	< 0.001	0.608
Mortality (%)^3^	17.50 ± 6.083	16.50 ± 9.330	10.44 ± 1.166	-	-	-

a,ab *P* = 0.097; ^a,b^*P* = 0.010; ^ab,b^*P* = 0.687.

1 Data: mean ± SD.

2 The analysis was performed with the mean of the performance of all individuals in each group over 4 wk; Control diet: n = 24; 1.5% BSFL meal diet: n = 24; 3.0% BSFL meal diet: n = 26.

3 The analysis was performed with three replicates in each dietary treatment.

### Egg Quality

The overall egg qualities, including egg weight, eggshell parameters, albumen weight, Haugh unit, and yolk parameters, are shown in Table 4. The average egg weights of the BSFL substitutional groups were higher (*P* < 0.05) than that of the control. There was no significant difference between the groups in terms of eggshell parameters, albumen weight, Haugh unit, yolk height, and yolk color. However, the yolk weight of the BSFL substitutional groups was higher (*P* < 0.01) than that of the control. The effect of the interval (4 wk) on egg quality was significant, but the effect of the interaction (interval and group) was not significant.

**Table 4:** Effect of dietary treatments with different proportion of BSFL meal substitution on egg quality^1^.

Egg quality	Control diet	1.5% BSFL meal diet	3.0% BSFL meal diet	*P*-values of effects		
				Group	Period (4 wk)	Groups*period
Egg weight (g)	56.59 ± 0.363^b^	58.20 ± 0.263^a^	59.16 ± 0.679^a^	0.002	< 0.001	0.140
Eggshell weight (g)	7.86 ± 0.346	7.85 ± 0.219	7.98 ± 0.291	0.514	< 0.001	0.493
Eggshell thickness (mm)	0.38 ± 0.010	0.38 ± 0.007	0.39 ± 0.007	0.344	< 0.001	0.858
Eggshell strength (kg/cm^2^)	4.27 ± 0.261	4.33 ± 0.154	4.39 ± 0.310	0.507	< 0.001	0.486
Albumen weight (g)	34.97 ± 1.289	34.83 ± 0.886	35.68 ± 1.000	0.072	< 0.001	0.618
Yolk height(mm)	7.53 ± 0.562	7.70 ± 0.202	7.54 ± 0.394	0.350	0.001	0.406
Yolk weight (g)	14.62 ± 0.460^c^	15.62 ± 0.468^b^	16.07 ± 0.391^a^	< 0.001	< 0.001	0.517
Yolk color	4.54 ± 0.468	4.19 ± 0.346	4.42 ± 0.336	0.156	< 0.001	0.970
Haugh unit	85.60 ± 3.915	87.40 ± 1.178	85.39 ± 1.873	0.188	< 0.001	0.265

Abbreviation: BSFL, Black Soldier Fly Larvae.

Mean values within a row with different superscripts letters represent the statistical differences.

a,b *P* < 0.05′; ^a,c; b,c^*P* < 0.01.

2 Data: mean ± SD, n = 12.

## DISCUSSION

The rearing systems of omnivorous BSFL may result in nutritional flexibility in the prepared BSFL meal. The crude protein portion of the BSFL meal used in the present study was 52.6%, which was in the range of reported crude protein in BSFL, but lower than that of BSFL meal reared on fish waste in a similar period (71.3%). The difference may attribute to the overestimation of crude protein in BSF meal reared on fish waste using a general conversion factor (6.25), which is proposed as 4.67 because of the presence of nonprotein nitrogen in BSFL, such as chitin (Janssen et al., 2017; Barroso et al*.*, 2019). In addition, instead of a full-fat BSFL meal (31–35%), the BSFL meal used in this study was moderately defatted (Diener et al., 2009) and had 15% crude fat content on a dry matter basis, which was higher than that in the fish meal. Although crude ash of the BSFL meal was lower than that of the fish meal, it met the general mineral requirements of poultry (Chia et al., 2020). Overall, the crude protein content of the defatted BSFL meal reared on house organic wastes was comparable to that of the fish meal, indicating that BSFL meal may be used as a protein source for substituting fish meal in poultry feed.

A balanced, rational amino acid inclusion is crucial to avoiding energy loss and improving performance. A previous study had indicated that the omission of even a single amino acid in the diet affected layers’ performance (Fisher and Johnson, 1956). In the present study, the amino acid composition of the BSFL meal was similar to that of the reported BSFL meal in the literature for most amino acids (Mwaniki et al*.*, 2018). Moreover, the BSFL meal showed a well-balanced amino acid composition, which was comparable to the fish meal; both were higher in glutamic acid and aspartic acid, and lower in cysteine and tryptophan. Although some studies suggest that low dietary lysine and methionine within the nutritional requirement may be positively correlated to feed intake, the BSFL diets with low lysine and methionine only had slightly higher feed intake than the fish meal group and without significant differences. Also, no abnormal behavior, such as tonic immobility, was found associated with high tyrosine in the BSFL diets (Macelline et al., 2021). Given this, BSFL meal may be feasible to formulate in poultry feed as an alternative to fish meal for amino acid supplementation. In addition, another study suggested that deficiency of amino acids in diets such as threonine and arginine may increase mortality via regulating oxidative stress and immune response (Alagawany et al., 2020). However, mortality of the BSFL substitutional group was not linearly associated with different dietary BSFL meal inclusions and was lower than that of the control, which may be explained by that all diets met layers’ nutritional requirements. The molecules in BSFL meal that may aid in lowering mortality remain unclear and need to be further studied (Lee et al., 2018).

In this study, the substitution of fish meal with BSFL meal did not result in any adverse effects on the laying performance of layers. The weekly transition of the laying rate in the BSFL substitutional group paralleled that of the control throughout the experiments and showed low laying rates around wk 8 and wk 40, which may be due to the temperature differences between winter and summer. Similar impacts were found in feed intake; the feed intake was higher around winter (to meet the energy requirement) and lesser around summer. The weekly transition trend of FCR was not similar to that of the laying rate and feed intake, which may owe to no effect of temperature on FCR when meeting basic nutritional requirements by the ad libitum diets. The higher feed intake in the groups fed with BSFL substitutional diets may suggest that BSFL meal was palatable to poultry (Wang and Shelomi, 2017). The laying hens’ performances were significant difference at 4-wk intervals. However, all groups did not show significant differences among different diets in terms of laying rate, feed intake and FCR. These results differed from the findings of Marono et al*.* (2017), who concluded that BSFL diets resulted in favorable FCR but low laying rate and feed intake, and Mwaniki et al*.* (2018), who concluded that 7.5% defatted BSFL diets significantly increased the feed intake and FCR value but had no effect on laying rate. The difference could be ascribed to the diet ingredients, bird age and strains. In this study, the 3.0% BSFL substitutional diets exhibited a positive effect on body weight, which might be due to high feed intake.

The nutritional value of the diets in terms of protein, total amino acids, and minerals was considered a key factor in egg quality. Several studies had indicated that calcium-rich BSFL diets resulted in eggshell quality improvement (Kawasaki et al*.*, 2019; Patterson et al., 2021). However, in the current study, eggshell quality, including weight, thickness, and breaking strength, did not differ significantly from the control. This discrepancy may be explained by the fact that using less BSFL meal in place of the fish meal had a poor effect on calcium and phosphorus levels which are involved in eggshell formation (Neijat et al., 2011). Additionally, the diet did not significantly affect the albumen weight, yolk color and height, and Haugh unit, which was in accordance with the observations of Heuel et al*.* (2021a). Interestingly, the results showed that the BSFL diets significantly increased the yolk weight. The major macronutrients in egg yolk are fat (∼26.7%) and protein (∼15.5%) (Réhault-Godbert et al*.*, 2019). Previously, it has been reported that the high level of fatty acids in BSFL meal, such as myristic and palmitic acid, could be transferred to the yolk, but they did not affect yolk weight (Heuel et al*.*, 2021b). In addition, Penz Júnior and Jensen. (1991) reported that the yolk weight of eggs was significantly lower when the layers were fed a 13% crude protein feed compared to a 16% crude protein feed. However, in this study, only the 3% BSFL diet had a slightly higher amount of crude protein content compared to the control. Thus, the high intake of BSFL diets possibly led to a high value in yolk weight and egg weight (Bouvarel et al*.*, 2011).

Taken together with the nutrients and effects of BSFL diets, BSFL meal could be an alternative to fish meal in feed formulation without nutritional deficiency risks. Moreover, the diets formulated with equal proportions of BSFL meal to fish meal can be isocaloric and isonitrogenous. However, the BSFL and fish meal diets in this study showed a different effect on the performance and production of laying hens, possibly since BSFL addition contributes to the modulation of nutritional metabolism and immune response. The hypothesis of BSFL dietary effect was confirmed in a study related to immunomodulatory of broilers and was considered as metabolites of major components in BSFL trigger the dietary effects (Lee et al*.*, 2018). The exact mechanism of the BSLF dietary effect is still in the process.

## CONCLUSIONS

In summary, the results of this study, which examined the effect of BSFL meal on the performance of laying hens (laying rate, feed intake, FCR, and body weight) and their egg quality (egg weight, eggshell parameters, albumen weight, yolk parameters, and Haugh unit) indicated that even if BSFL were fed leftovers and the meal was defatted with organic solvents, it can be an ingredient of poultry feed without any adverse effects. The complete substitution of fish meal with BSFL meal could be a feasible way to contribute effectively to the performance of layers. However, the mechanisms underlying the increase in body weight and yolk weight are not clear yet. Further studies in this area should focus on the changes in the gut microbiota and amino acid metabolism.
